# Divergent patterns of selection on metabolite levels and gene expression

**DOI:** 10.1186/s12862-021-01915-5

**Published:** 2021-09-29

**Authors:** Alexander F. Kern, Grace Xiaolu Yang, Neil M. Khosla, Roy Moh Lik Ang, Michael P. Snyder, Hunter B. Fraser

**Affiliations:** 1 Department of Genetics, Stanford University, Stanford, CA USA; 2 Department of Biology, Stanford University, Stanford, CA USA

**Keywords:** Polygenic adaptation, Metabolism, Gene expression, Yeast, Ergosterol, Stabilizing selection, Metabolic evolution

## Abstract

**Background:**

Natural selection can act on multiple genes in the same pathway, leading to polygenic adaptation. For example, adaptive changes were found to down-regulate six genes involved in ergosterol biosynthesis—an essential pathway targeted by many antifungal drugs—in some strains of the yeast *Saccharomyces cerevisiae*. However, the impact of this polygenic adaptation on metabolite levels was unknown. Here, we performed targeted mass spectrometry to measure the levels of eight metabolites in this pathway in 74 yeast strains from a genetic cross.

**Results:**

Through quantitative trait locus (QTL) mapping we identified 19 loci affecting ergosterol pathway metabolite levels, many of which overlap loci that also impact gene expression within the pathway. We then used the recently developed v-test, which identified selection acting upon three metabolite levels within the pathway, none of which were predictable from the gene expression adaptation.

**Conclusions:**

These data showed that effects of selection on metabolite levels were complex and not predictable from gene expression data. This suggests that a deeper understanding of metabolism is necessary before we can understand the impacts of even relatively straightforward gene expression adaptations on metabolic pathways.

**Supplementary Information:**

The online version contains supplementary material available at 10.1186/s12862-021-01915-5.

## Background

Natural selection acting on diverse traits and genomic loci has been identified in many organisms and characterized at molecular, genetic, and phenotypic levels [1–3]. Although several clear examples of single-locus adaptations of large effect have been identified [1, 3, 4], there is mounting evidence that most adaptation occurs through many variants of small effect, resulting in highly polygenic trait architectures [5–7]. Understanding these complex adaptations is of key importance in evolutionary biology, but remains difficult because small effect loci are challenging to detect via traditional methods such as quantitative trait locus (QTL) mapping.

One alternative approach is the sign test, which aims to identify groups of genes where selection has led to up- or down-regulation via independent mutations. First, the *cis*-regulatory divergence between two species is quantified genome-wide, typically via allele-specific expression (ASE) analysis in an F_1_ hybrid [8–10]. This results in directionality information for every gene (e.g., the species A allele is up-regulated compared to the B allele, meaning it produces more copies of mRNA). Any group of genes not under directional selection should have a frequency of A allele up-regulation similar to that of the entire genome. By contrast, if 50% of genes genome-wide have A allele up-regulation, but a significant majority of genes in a particular pathway have their A alleles up-regulated, this indicates the action of lineage-specific natural selection [9]. The sign test is most powerful when many genes are involved, making it uniquely well-suited for studying polygenic adaptations.

The first application of the sign test to gene expression data identified the ergosterol biosynthesis pathway as a target of recent adaptation in the budding yeast *Saccharomyces cerevisiae* [11]. Specifically, six genes clustered in the late steps of the pathway all showed down-regulation in a laboratory strain (BY) as compared to a wild isolate (RM). Further characterization of this adaptation revealed several key details: (1) down-regulation was due to a combination of *cis*-acting effects specific to each gene as well as *trans*-acting effects from a transposon insertion in the transcription factor HAP1, (2) a deficit of genetic polymorphisms specifically at the 5’ ends of these six genes supported the sign test’s evidence of selective sweeps; (3) these selective sweeps occurred quite recently (e.g., in the last few decades for HAP1); (4) Most sweeps involved standing variation that is common in many strains of *S. cerevisiae*; (5) At one target gene, *ERG28*, the causal mutation was found to be a 2-bp deletion in the promoter that disrupted binding of two transcription factors [12], (6) this mutation is only advantageous in certain environments (i.e., condition-specific).

However, even in this relatively well-studied example, we know nothing about the effects of this coordinated down-regulation beyond the mRNA level. A critical question is how these effects propagate to affect metabolite levels. Since enzymes and metabolites within this pathway are targeted by many widely used antifungal drugs [13], the late ergosterol biosynthesis pathway has been exceptionally well-studied, making it an ideal test case for understanding adaptation. For instance, there is extensive regulation at the levels of protein degradation and localization in *S. cerevisiae* [14]. Proteasomal degradation of these enzymes has been observed in response to the levels of other metabolites [15], mislocalization [16], or unknown causes [17]. Numerous other regulatory mechanisms act upon this pathway as well. Taking *ERG1* as an example, its activity is regulated by its subcellular localization between lipid particles and the endoplasmic reticulum [18], by lanosterol levels through proteasomal degradation [15], and by iron, oxygen, and sterol levels through transcriptional regulation from *UPC2* and *ECM22*, which are in turn regulated by *HAP1* and *MOT3* [19–21]. In addition, sterol levels are regulated directly through export, esterification, and acetylation to prevent toxic build-up [22]. Notably, many of these regulatory mechanisms occur post-transcriptionally, and serve to tune metabolite levels to the environment. The complexity of this multi-layered regulation highlights the importance of directly measuring metabolite levels to more fully characterize selection acting upon the pathway.

Previous studies have sought to link genetic variation at the gene expression level to variation at the metabolite level (e.g. [23–27]. For instance, a study using an *Arabidopsis* Bay × Sha cross was used to map metabolite and eQTL for the aliphatic and indolic glucosinolate pathways, and determined that all eQTL for the pathway overlapped metabolite QTL, but the reciprocal was not true [28]. This study also identified epistasis and transgressive segregation in which some segregants had metabolite levels higher or lower than both of the parents within these pathways [28]. There have been several studies examining this link in the *S. cerevisiae* BY x RM cross used in this study as well [29, 30]. These studies examined several metabolite levels, none of which were in the ergosterol biosynthesis pathway, and identified overlapping hotspots between eQTL and metabolite QTL including at *IRA2* and *HAP1*, and also observed transgressive segregation for several metabolites. These studies and others have been invaluable in linking genetic variation in metabolite levels and gene expression, but none have examined the effect of a polygenic gene expression adaptation within a pathway on metabolite levels, which may be expected to have a profound effect.

In this work, we sought to ask what the effects of polygenic gene expression adaptation are in a well-studied, linear metabolic pathway. The six down-regulated genes are localized to a section of the pathway removed from any known branch points where selection on branch point enzymes can redirect flux through productive alternative branches [31]. Though predicting metabolic pathway activity through metabolite QTL or eQTL has been shown to be difficult [32], this polygenic downregulation may be expected to have a more predictable and widespread affect, akin to a strong gene knockdown. Therefore, a reasonable and parsimonious expectation could be that the adaptation would yield lower flux through the down-regulated section of the pathway (akin to a traffic jam), leading to build-up of precursors and lower levels of downstream products including ergosterol. However, our results show that the divergence in metabolite levels is more complex, and presently unpredictable from patterns of gene expression.

## Results

**Fig. 1:**
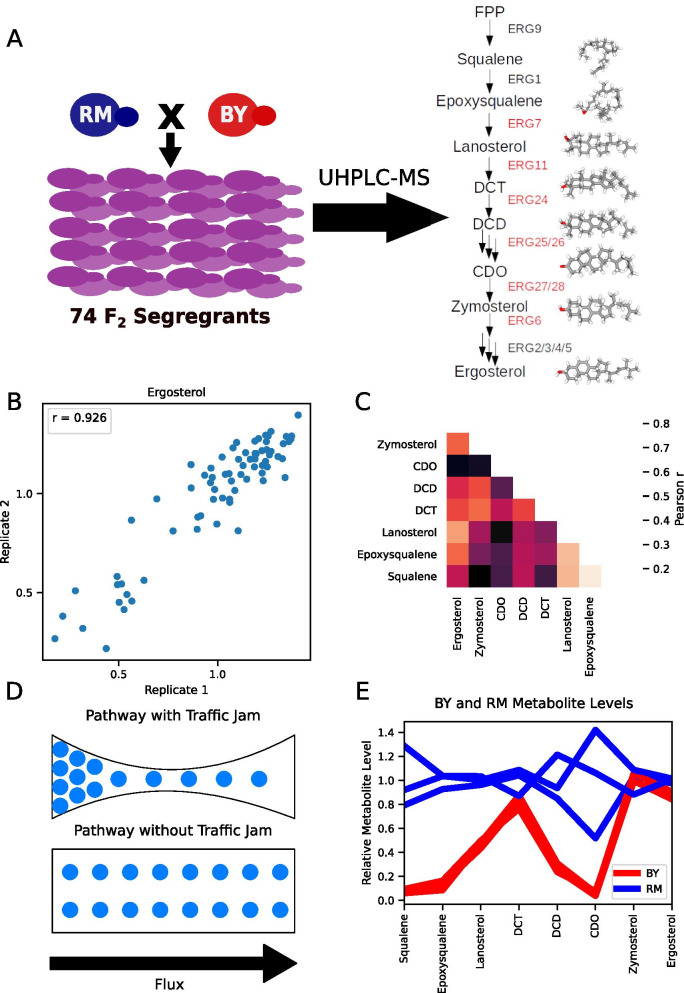
Metabolic Profiling of F2 haploid segregants from a cross between BY and RM. **A** Eight metabolites produced in the late ergosterol biosynthesis pathway were profiled using targeted metabolomics with UHPLC-MS in 74 haploid F2 segregants from a cross between the BY (laboratory) and RM (wine) strains (genes previously shown to be under selection in [11] for gene expression in red). Structures shown for metabolites measured. **B** Between technical replicate pearson correlation of ergosterol level for 73 F2 segregants with technical replicates, metabolite levels scaled by the mean normalized peak area for the segregants. **C** Correlations between the different measured metabolite levels for the segregants (mean of technical replicates). **D** Diagram depicting the traffic jam model in which a reduction in enzyme levels along a section of a linear pathway leads to build up of precursor metabolites and reduction in pathway end products, as well as an unobstructed metabolic pathway. **E** Metabolite levels for the three biological replicates of BY and RM scaled to the mean of the three RM biological replicates

To characterize how selection at the level of gene expression has impacted metabolite levels in the late ergosterol biosynthesis pathway, we utilized segregants from a well-characterized cross between two strains of yeast: BY, a laboratory strain, and RM, a wine strain. F_2_ haploid segregants from this cross have been profiled for genome-wide gene expression [33], protein expression [34], cellular morphology [35], and growth on dozens of different substrates [36]. We used ultra-high-performance liquid chromatography-tandem mass spectrometry (UHPLC-MS) to profile eight metabolites (Additional file 6: Table S1) in the late ergosterol biosynthesis pathway for the BY and RM strains, as well as 74 of their segregants (Fig. 1A). Metabolite levels showed generally high correlations between technical replicates where samples from the same culture were measured twice, with a few outliers (Fig. 1B, Additional file 1: Fig. S1). Metabolite levels for the same strains were well correlated within the first two steps of the pathway, but further downstream the order of the metabolites within the linear pathway did not predict the correlation between the metabolite levels, reflecting the complex regulation of the pathway (Fig. 1C).

The metabolite data from BY and RM parental strains immediately disproved some simple predictions from the gene expression differences. Based on the polygenic down-regulation of the enzyme-encoding genes from *ERG7* to *ERG6* in the BY strain, one might expect a “traffic jam” caused by this bottleneck in the pathway (Fig. 1D). More specifically, this model would predict lower metabolite levels in BY starting at the product of the first enzyme affected by the down-regulation, and higher substrate levels earlier in the pathway, due to the lower flux through the bottleneck caused by a series of down-regulated enzymes. However, we did not observe this pattern: the levels of zymosterol are extremely similar between BY and RM, and the squalene levels upstream of the polygenic downregulation are much higher in RM—the opposite of the traffic jam model’s prediction (Fig. 1E). While our data are not consistent with the traffic jam model, we also note that our data do not allow us to infer pathway flux, since we are relying on static metabolite measurements rather than direct measurements of flux. For example, RM could produce more ergosterol, but also export it much more quickly, leading to lower steady-state levels.

**Fig. 2:**
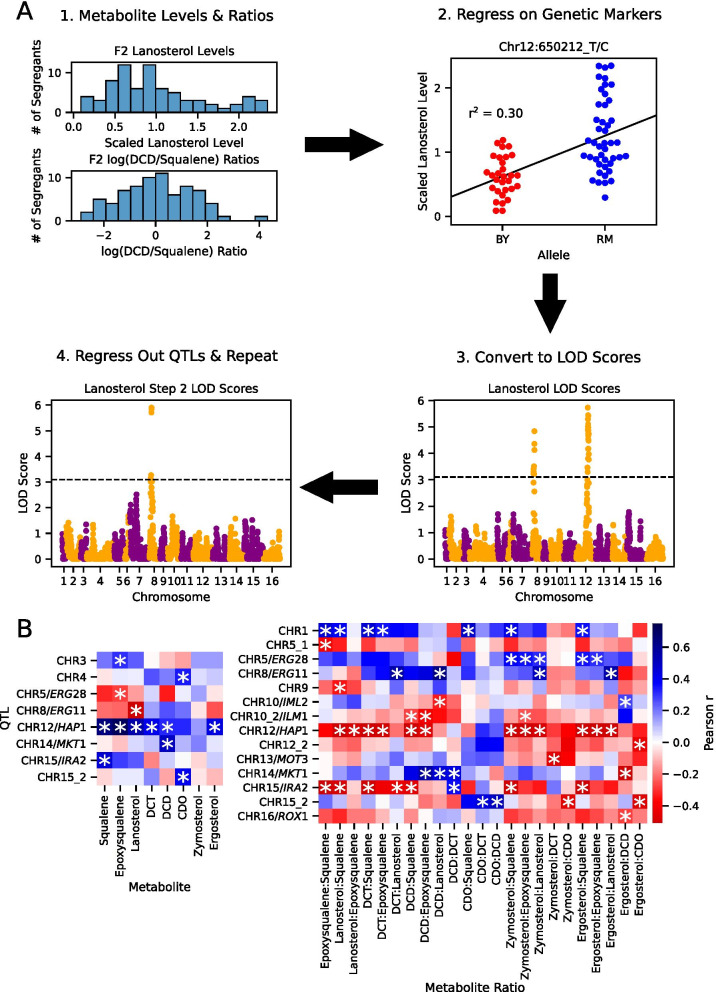
Metabolite QTL Mapping. **A** The QTL mapping process for this study: **1**) Histograms showing the lanosterol and log(DCD/Squalene levels) for the F2 segregants. **2**) lanosterol levels for F2 segregants, split based on their allele (BY or RM) at the peak marker variant for the Chr 12/HAP1 QTL. Pearson r^2^ for the marker correlation with lanosterol level scaled to the mean of the segregant levels shown. **3**) LOD score plot for the first round of QTL mapping for lanosterol levels. **4**) LOD score plot for the second round of QTL mapping after regressing out the QTL mapped in the first round. **B** Pearson correlations between the peak marker for each QTL and metabolite levels and ratios. Separate heatmaps are shown for QTL mapped using metabolite levels and metabolite ratios. Red indicates segregants with the BY allele have higher levels of the metabolite or ratio, and blue indicates segregants with the RM allele have higher levels of the metabolite or ratio. QTLs which are significant for a given metabolite level or ratio are marked with * in the cell matching the row of the QTL and the column of the metabolite or ratio which it affects

To determine the segregating genetic loci affecting the late ergosterol pathway metabolite levels, we mapped quantitative trait loci (QTL) using both absolute metabolite levels and their pairwise log ratios as quantitative traits (Fig. 2A, Additional files 2, 9: Fig. S2). We utilized a previously published forward-stepwise regression approach to map QTL [37, 38]. Briefly, each segregant’s genotypes at loci differing between the two strains were regressed on metabolite levels and ratios. We then use forward stepwise regression, where QTL from previous rounds of regression were added to the linear model to remove their effects and increase power to map additional QTL (Fig. 2A). Although our QTL did not have sufficient resolution to pinpoint individual genes (the median 1.5-LOD interval contained 26 genes), we did observe many of the genes involved in the ergosterol pathway polygenic adaptation within QTL intervals. For example, we identified QTL containing *ERG11*, *ERG28*, and *HAP1* (Fig. 2B). Overall we identified 8 unique QTL for metabolite ratios alone, 6 QTL for both metabolite levels and ratios, and 2 for metabolite levels alone (LOD score cutoffs were identified for each metabolite and ratio via permutation (LOD cutoff range = 2.52–3.19, GWER_1_ = 0.10). Several of the QTL were significant for multiple ratios and metabolite levels.

Having mapped metabolite level QTL, we then asked whether they were consistent with expectations based on previously mapped eQTL mapped using a larger panel of the same cross and similar methodology [33]. Notably, 8/16 QTL mapped for metabolite levels and ratios overlapped with previously mapped eQTL for genes within the ergosterol biosynthesis pathway, compared to ~ 4 expected by chance (permutation p-value = 0.0443, Additional file 3: Fig S3). Due to the difference in the number of strains used for QTL mapping for the metabolite traits versus the eQTL (74 vs. 1012), there is a substantial difference in power, and so with additional power more metabolite QTL overlapping eQTL may be revealed. For example, lanosterol levels were most strongly affected by two loci on chromosomes 12 and 8, containing *HAP1* and *ERG11* (Fig. 2B). This makes sense considering that lanosterol is the substrate for Erg11p, and the two strongest eQTL for *ERG11* are its local (likely cis-acting) genotype and the trans-acting *HAP1* genotype [33]. However, although the RM alleles at both of these eQTL increase *ERG11* mRNA levels, they had opposite effects on lanosterol levels, with the RM allele increasing lanosterol at *HAP1* but decreasing lanosterol at *ERG11* (Fig. 2B). Naively one would expect increasing levels of an enzyme to decrease levels of its substrate, yet the *HAP1* QTL contradicts this expectation. This is less puzzling when considering that the *HAP1* QTL affects the levels of many enzymes in this pathway in addition to *ERG11*, illustrating one aspect of the difficulty in extrapolating from expression levels to metabolite levels.

We next asked if the patterns of selection acting on metabolite levels may be predictable from those previously reported on expression levels. One approach to this would be to detect selection acting on metabolite levels using the QTL sign test. Unfortunately, even if all of the QTL affect the trait in the same direction, this test requires 8 QTL to achieve a probability less than 0.01 (p = (½)^8–1^). Since we were not able to identify that number of QTL for any of the metabolite levels or ratios individually, we were underpowered to test for selection using this approach.

We recently developed an alternative selection test (the v-test) that can be more powerful than the sign test when insufficient numbers of QTLs are mapped [39]. In this test the F_2_ segregant phenotypic distribution is treated as a null model for potential parental phenotypes expected under neutral evolution. While the parents, as extant strains, have been subject to selection over many generations, the segregants have not (with the exception of selection for viability in rich media), and thus represent the distribution of possible phenotypic states given the genetic variation present in the two parents. In short, the F_2_ phenotypic distribution represents a randomization of the genetic variants affecting a trait. If the true phenotypic difference between the parental strains is much larger than expected from this null distribution, it indicates that the genetic variants affecting this trait are distributed non-randomly in the parents to make them more divergent than expected by chance (i.e., diversifying selection). If the parental difference is smaller (i.e.*,* transgressive segregation), it suggests stabilizing selection has acted on that trait in the parents, since the genetic variants affecting this trait are distributed non-randomly so that the two parental trait values are more similar than expected.

Our application of the v-test framework to late ergosterol biosynthesis pathway metabolite levels identified two metabolites (epoxysqualene and DCD) with a phenotypic pattern suggestive of directional selection and one (zymosterol) with evidence of stabilizing selection. However, we noticed that the data for the F_2_ trait distributions violated a requirement of the v-test for normally distributed trait values (see “Methods”).

**Fig. 3:**
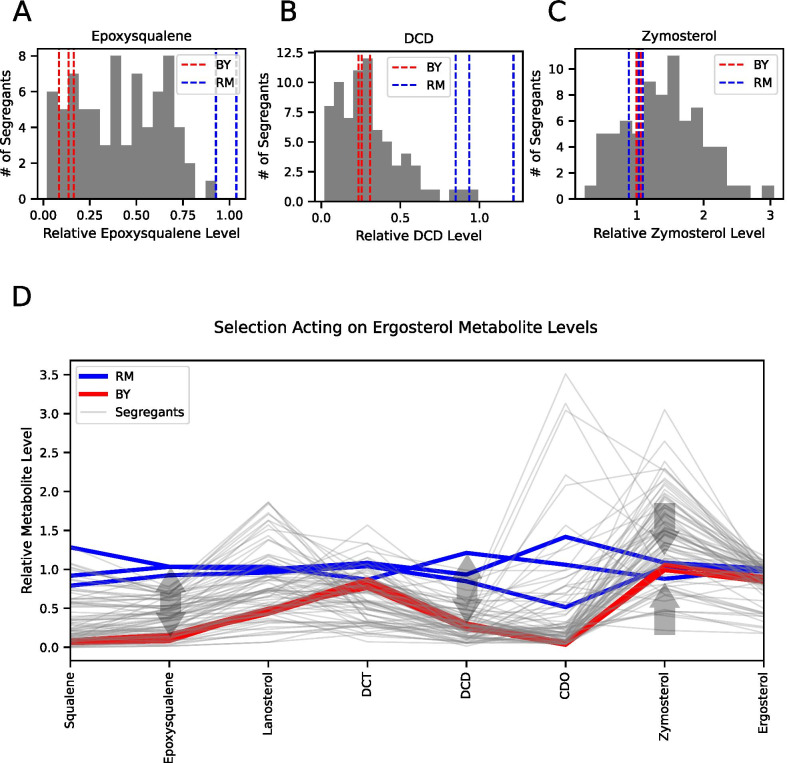
Phenotypic distributions of parents and segregants show selection acting in different directions at multiple steps in the pathway. **A** Histogram of F2 segregants’ distribution of epoxysqualene levels, with three biological replicate measurements of each of the parental strains (BY red, RM blue). **B** Histogram of segregants’ distribution of DCD levels, with three biological replicate measurements of each of the parental strains (BY red, RM blue). **C** Histogram of segregants’ distribution of zymosterol levels, with three biological replicate measurements of each of the parental strains (BY red, RM blue). **D** Line graph showing segregant and parental replicate trajectories along the ergosterol pathway. Arrows indicate the type of selection acting on the parents at steps where selection was detected

We therefore developed a non-parametric version of the test based on comparing trait dispersions in parents vs F_2_. We determined the segregant’s dispersions by comparing their metabolite levels to the mean level, and the parental dispersions by comparing each of the three BY biological replicates’ metabolite levels to the mean of it and one of the RM replicates, giving us three independent measurements of the parental metabolite dispersions. We then compared the segregant and parental distributions for each metabolite to see whether the parental dispersions were significantly higher or lower than the segregant dispersions using the Kruskal–Wallis test. To assess significance of this test, we used 20,000 permutations to determine the empirical p-value distribution of the test statistic (see “Methods”). The results of this test implicate the same three steps of the pathway with significant differences between the parent and segregant distributions. Specifically, epoxysqualene (all sets p ≤ 0.0192 after Bonferroni correction) and DCD levels (all sets p ≤ 0.0944 after Bonferroni correction) showed evidence of directional selection (Fig. 3A and B). In contrast, zymosterol (all sets p ≤ 0.0272 after Bonferroni correction; Fig. 3C) showed evidence of stabilizing selection, where the parental levels were more similar than expected. Thus, different metabolites in the ergosterol pathway are evolving under different types of selection (Fig. 3D).

In addition to the differences in dispersion between parental and segregant metabolite levels, several of the metabolite levels also showed differences in mean, which is indicative of epistasis. We calculated the Δ- statistic [40] and tested for epistasis using it via a permutation approach [41]. This test identified epoxysqualene, DCT, DCD, and zymosterol as showing significant evidence of epistasis at a Bonferroni-corrected p-value threshold of 0.05. Interestingly, all of the metabolites identified as being under selection by the v-test also showed evidence of epistasis.

## Discussion

In this paper, we examined selection acting on metabolite levels in the ergosterol biosynthesis pathway between two well-studied strains of baker’s yeast. We found several QTL affecting metabolite levels and ratios, half of which have been previously identified as eQTL affecting genes in the pathway. Increasing the number of segregants measured could potentially identify additional metabolite QTL, which may overlap a greater fraction of the eQTL, but it is less likely that the metabolite QTL not overlapping eQTL would be covered, due to the much higher power for the eQTL mapping [33].

We also identified three metabolite levels under selection: epoxysqualene, DCD, and zymosterol. Interestingly, selection appeared to be affecting these steps differently: diversifying selection is likely acting on epoxysqualene and DCD levels, whereas stabilizing selection is likely acting on zymosterol. As shown by the correlation between metabolite levels and the many shared QTL for this pathway, ergosterol pathway metabolite levels show high levels of genetic correlation. Thus, though the phenotypic patterns of selection on epoxysqualene and zymosterol show that they have been affected by selection, this does not imply that they are the direct targets of selection; it is possible that selection is acting on only one of these traits or another unmeasured trait. Nonetheless, this was surprising given previous work showing polygenic downregulation of six genes in this pathway in the BY strain relative to RM. Thus, we have presented evidence against the simple expectation from the gene expression results that the pathway may have been selected for lower activity in BY.

There are many possible reasons for why the models from the polygenic downregulation and the metabolite levels do not seem to match up. One reason could be due to protein sequence changes within these genes between BY and RM. There are eight total coding changes between BY and RM within all of the genes shown in Fig. 1; eight out of fourteen of these genes had no coding differences, including six out of the eight genes within the polygenic downregulation (highlighted in red). Another possible mechanism for this unexpected result could be that other genes in the pathway are upregulated or have differences in their expression which could compensate for the polygenic downregulation. However, there was no clear compensatory upregulation of other genes within the ergosterol biosynthesis pathway within BY, as all genes within the ergosterol biosynthetic pathway besides *NCP1* and *ERG3* were more highly expressed in RM than in BY [33]. Another possibility could be changes in translational efficiency or post-translational modifications to the enzymes in this pathway between BY and RM, which is an exciting question for future study. Substantial changes in esterification, sequestration, import, and export of metabolites from this pathway could also contribute to these results and would also be a good topic for future study. One striking example of this possibility is that partitioning of Hmg1p to nucleus-vacuole junctions, even without any change in protein level, increases flux through the mevalonate and ergosterol biosynthesis pathways [42]. The epistasis identified for several of the metabolite levels may also contribute to this complexity. In concert with these processes, there is extensive feedback regulation of the ergosterol pathway at both transcriptional and post-transcriptional levels that likely contributes to the complexity of predicting metabolite levels, though feedback at the transcriptional level should be visible in the gene expression levels analyzed. Study of metabolic flux, which we can not determine from steady state metabolite levels, may elucidate some of these factors. These results highlight the value of measuring metabolite level data, as our naive expectations from gene expression were inaccurate, and the patterns of selection we identified would have been difficult to predict from gene expression data alone.

While it is difficult to connect selection acting on ergosterol pathway metabolite levels to organismal phenotypes such as response to certain environments or to fitness, previous work on this pathway helps to point to some connections. Zymosterol is the first metabolite in the pathway that can functionally replace ergosterol and maintain cell viability [43], which makes our observation of stabilizing selection on zymosterol (Fig. 3C and D) particularly interesting. By maintaining nearly constant levels of zymosterol while reducing the potentially toxic levels of upstream metabolites such as epoxysqualene and DCD [22], the BY strain’s adaptation may be an effective means to maintain pathway output while reducing toxicity. In addition, previous study of the causal variant underlying the cis-regulatory expression differences between BY and RM in ERG28 found that the variant increased resistance to the antifungal Amphotericin B [12]. Allowing for increased antifungal resistance while maintaining the production of functional sterols could be beneficial for fitness in varying environments. This balancing act is in contrast to non-essential metabolic pathways, which have been shown to evolve between species largely through reductive evolution in budding yeasts [44].

Although selection on ergosterol biosynthesis between these two strains may have occurred in the BY lineage after its introduction to the laboratory, there has been no deliberate selection on this trait (to the best of our knowledge). Thus, this pathway may serve as a useful model for natural selection acting on metabolic pathways more generally.

## Conclusions

Overall, our results suggest that patterns of selection on metabolite levels are not easily predictable from selection on gene expression. Even a seemingly simple polygenic downregulation, in which several adjacent genes in a pathway are downregulated in one strain relative to another, did not allow for simple prediction of the effects of the selection on metabolite levels. This underscores the importance of studying selection at multiple levels of molecular phenotypes, and particularly those more directly affecting phenotypes contributing to fitness. Further studies of this type will help to shed light on how changes in the transcriptome impact the metabolome, and contribute to our understanding of the evolution and genetic basis of complex traits.

## Materials and methods

### Yeast strains

We used 74 meiotic segregants previously generated in [37] from a cross between the prototrophic yeast laboratory strain BY (*MATa,* derived from a cross between BY4716 and BY4700) and the prototrophic vineyard strain RM (*MATα hoΔ::*hphMX4 *flo8*Δ::natMX4 *AMN1-BY*; derived from RM11-1a), as well as the parental strains BY and RM.

### Yeast growth and preparation for metabolite extraction

All segregants and parental strains were inoculated from glycerol stocks into 2 mL of standard synthetic complete media (SCM) (Yeast Nitrogen Base with Ammonium Sulfate (Fisher Cat. #50-489-163), Dropout Mix Complete (US Biological Cat. #D9515), and 2% glucose) in 14 mL Falcon™ round-bottom culture tubes (Falcon™ 352059) and grown overnight, shaking at 30 °C.

All overnight cultures were measured for their optical density (OD) and recorded. These measurements were used to determine the volume of overnight culture needed to inoculate a 30 ml flask of SCM to a starting OD of approximately 0.3. Flasks were then incubated, shaking, at 30 °C for 3–4 h until their OD reached approximately 0.6–0.65.

Flasks were then removed, and their OD was measured and recorded. 21 mL of culture from each flask was transferred to 50 mL Falcon® conical tubes (Corning 352070), and centrifuged at 3000 rpm at 4 °C. Supernatant was discarded and pellet was resuspended in 2 mL of MS-grade H2O, mixed by gentle pipetting, and 1 mL was distributed to each of two screw-cap tubes (Bio Plas 4202SLS). Tubes were then centrifuged at 3000 rpm at 4 °C to pellet yeast.

Supernatant was discarded, and pellet was left as dry as possible. Pellets were immediately frozen at −80 °C in preparation for metabolite extraction. Information on strain growth was recorded (Additional file 11).

### Metabolite extraction

Chilled Lysing Matrix C beads (MP Biomedicals) were added to frozen cell pellets on dry ice. Chilled methanol was then added to the tube containing beads and pellets. Samples were disrupted on FastPrep-24™ Benchtop Homogenizer (MP Biomedicals) and centrifuged at maximum speed for 10 min. Supernatant was transferred into an Eppendorf® Protein LoBind Tube and dried using a TurboVap® Evaporator (Biotage). The evaporated samples were reconstituted in methanol, vortexed briefly, centrifuged, and the supernatant was collected in an amber screw-top vial (Waters). The metabolite extract was analyzed immediately on LC–MS or stored temporarily at −20 °C prior to analysis.

### LC–MS analytical methods

Targeted metabolite quantification was performed on a 1260 Infinity UHPLC coupled with a G6538A UHD Accurate Mass Q-TOF Mass Spectrometer (Agilent). UHPLC-MS conditions were optimized in terms of peak shape, reproducibility and retention times of different metabolites analyzed.

Chromatography was performed using an Acquity UPLC BEH Phenyl Column (Waters, 130 Å, 1.7 µm, 2.1 mm × 50 mm) kept at 60 °C. Separation was performed using gradient elution with 0.05% (v/v) acetic acid in 50%/50% methanol/water (A) and 0.05% (v/v) acetic acid in 100%/0% methanol/water (B) at a flow rate of 0.5 mL/min. Starting conditions were 100% A and 0% B for 1 min, changing non-linearly to 95% B over the next 15 min, followed by re-equilibration for 4 min prior to the next injection. Mass spectrometry analysis was performed in positive atmospheric pressure chemical ionization (APCI+) mode. Gas temperature was 350 °C, vaporizer temperature was 450 °C, capillary voltage was set at 3.5 kV, and drying gas flow rate was 9 L/min.

For each yeast strain, two biological replicates were analyzed. QC samples were also analyzed per 15 injections. For each injection, 5 µL of sample was injected into LC–MS. Data was collected in centroid mode with a scan range of 50–1000 m/z and acquisition rate of 1.5 spectra/s. Reference Mass Solution (Agilent) was injected at a flow rate of 0.4 mL/min and reference mass correction was enabled to perform mass correction.

### LC–MS data processing

LC–MS data was converted into the mzXML format and was processed using MZmine 2.33 [45], employing targeted peak picking and aligning. The ion intensities for each targeted peak were then normalized within each sample to the sum of all the peak intensities in that sample. The generated peak tables were exported for further analysis (Additional files 8, 9, 12).

### QTL mapping

QTL were mapped using normalized peak intensities for each segregant. Segregating genetic markers coded as −1 and 1 between the set of segregants used in this study were determined using genetic marker data on these strains from Albert et al. [33] (Additional file 7). Normalized metabolite peak levels for the segregants were regressed on each segregating genetic marker, and their log-odds (LOD) score was determined using the formula -number of segregants*log(1−r^2^)/(2*log(10)), where r is the Pearson correlation between the marker and the metabolite level. In addition, all unique pairwise ratios of metabolites were determined, and the log2ratio values were regressed upon segregating genetic markers, and LOD scores were determined as described above. To determine significance, each metabolite level or ratio was permuted 2000 times with respect to the genetic markers, and LOD scores were calculated on this permuted data. For genome-wide error rate control, the second highest LOD score among each permutation was taken, and the 90th percentile of these values was taken to provide a cutoff LOD score for a GWER_1_ < 0.10 [36, 38]. In each round of QTL mapping, only the maximum LOD score marker was taken, and kept as a QTL if it passed the LOD threshold, to prevent the possibility of shadow QTL [46]. To map additional QTL, the significant QTL from the previous round of regression was regressed out of the metabolite levels, and the residuals were used for mapping using the same protocol as described above. This was repeated, regressing out all QTL from previous rounds, until no additional QTL were mapped in a given round for a given metabolite. Boundaries of QTL were defined by a 1.5 LOD drop from the peak LOD, and expanded to all perfectly linked markers with these boundaries. For one segregant, one technical replicate had values far outside of the range of the rest of the strains for squalene and epoxysqualene (Additional file 1: Fig S1). For this segregant, the technical replicate whose values fell within the range of the rest of the segregants was used for QTL mapping for these metabolites. In addition, there were three segregants (including the one mentioned above) whose CDO levels were off diagonal on the technical replicate correlation plots. For each of these segregants, one replicate was chosen to use for the QTL mapping, based on having higher average correlation between metabolites.

### Permutation test for metabolite QTL and eQTL overlap

Metabolite QTL were collapsed such that any overlapping QTL were treated as a single QTL, with the narrowest possible QTL boundaries, due to the assumption that they represented the same causal locus (maximum lower confidence interval position, minimum higher confidence interval position). This yielded sixteen total metabolite QTL. Metabolite QTL lengths were kept constant, and their positions along the genome were permuted 10,000 times, ensuring that none of the permuted QTL spanned multiple chromosomes, and none of the permuted QTL overlapped. All eQTL from [33] for the 24 genes within the GO term GO:0006696: ergosterol biosynthetic process [47, 48] were obtained. If the peak marker for any of these eQTL was within the boundaries of a permuted metabolite QTL range, this was treated as an overlap. The number of permuted metabolite QTL containing a peak marker for any eQTL was recorded for each permutation. The permutation p-value was obtained by calculating the fraction of permutations with as many or more metabolite QTL overlaps than in the unpermuted data.

### V-test

The v-test was performed as described in Eq. 2 of Fraser [39] for all of the traits. The heritability (H^2^) of the segregants was calculated as the difference between the variance of the F2 segregants and the variance of the parents, scaled by the variance F2 segregants. Notably, the technical and environmental variation in the parental zymosterol measurements was larger than the biological variation, making it impossible to correct for the parental heritability, and so only the heritability within the F2 segregants was corrected for, which makes the test more conservative in the case of stabilizing selection. The DCD and epoxysqualene metabolite level F2 distributions were tested for normality using the Shapiro-Wilks test, and both deviated significantly from normality (DCD p = 3.73 × 10^–5^, epoxysqualene p = 0.025). Due to these violations of the v-test assumptions, we developed a non-parametric version of the test. For one segregant, one technical replicate had values far outside of the range of the rest of the strains for squalene and epoxysqualene (Additional file 1: Fig S1). For this segregant, the technical replicate whose values fell within the range of the rest of the segregants was used for the v-test for these metabolites. In addition, there were three segregants (including the one mentioned above) whose CDO levels were off diagonal on the technical replicate correlation plots. For each of these segregants, one replicate was chosen to use for the v-test, based on having higher average correlation between metabolites.

### Non-parametric metabolite level selection test

We first calculated the absolute difference between each segregant’s metabolite level and the mean level of the F_2_ segregants for each trait to determine the F_2_ segregants’ spread. Next, to determine the parental dispersion for each metabolite, we calculated the absolute difference in metabolite level between each BY biological replicate and the mean of that BY replicate and an RM replicate, using the mean of the two technical replicates for each of these measurements as we did for the segregants. This led to three independent measurements of dispersion for the parents from the three biological replicates each of BY and RM with no replicate being used in more than one comparison. Since there were multiple ways to pair the three BY and RM replicates, we repeated this procedure with three possible pairings of BY and RM biological replicates with no pairings repeated in separate tests (set 1: BY1/RM1, BY2/RM2, BY3/RM3; set 2: BY1/RM2, BY2/RM3, BY3/RM1; set3: BY1/RM3, BY2/RM1, BY3/RM2—numbering arbitrary) to ensure the results were consistent regardless of the choice of replicate pairings. We then compared the segregant and parental distributions for each metabolite using the Kruskal–Wallis test, a non-parametric test which allows us to determine whether parental spread values are significantly higher or lower than the segregant spread values (Additional file 5: Fig S5). The p-values from the Kruskal–Wallis test were not well-calibrated for many of the metabolites based on permutations (Additional files 4, 5: Fig S4, S5), possibly due to the unequal sample sizes of the parent sets (three) and the segregants (74). Thus, to assess the significance of this test, we used a permutation approach, wherein we randomly selected 6 strains’ metabolite levels from the combined set of parents and segregants, split them into two groups of three to represent the three parental replicates, and then obtained the Kruskal–Wallis test statistic as described above, using the six randomly chosen strains instead of the true parental strains. We repeated this procedure 20,000 times to get a permutation test statistic distribution. We then calculated the adjusted permutation p-values for each trait by counting the number of permutation test statistics greater than or equal to the actual test statisic for the parent-segregant comparison, dividing by 20,000, and multiplying by eight to perform Bonferroni multiple-test correction for tests on the eight metabolites. The highest corrected p-value is reported in the text, and for all three traits identified as being under selection, all of the parent sets showed significant corrected p-values at alpha = 0.10 (zymosterol p = 0.0048, 0.0272, 0.006; DCD p = 0.082, 0.0684, 0.096; epoxysqualene p = 0.0124, 0.0048, 0.0048). For one segregant, one technical replicate had values far outside of the range of the rest of the strains for squalene and epoxysqualene (Additional file 1: Fig S1). For this segregant, the technical replicate whose values fell within the range of the rest of the segregants was used for selection tests for these metabolites. In addition, there were three segregants (including the one mentioned above) whose CDO levels were off diagonal on the technical replicate correlation plots. For each of these segregants, one replicate was chosen to use for selection tests, based on having higher average correlation between metabolites.

### Epistasis test

The epistasis test was performed as described in [41]. Briefly, the Δ- statistic was calculated as $\Delta = \mu_{F2} - \frac{{\mu_{BY} + \mu_{RM} }}{2}$, where μ_F2_ is the mean of the F2 segregants, μ_BY_ is the mean of the BY replicates, and μ_RM_ is the mean of the RM replicates. The squared standard error of the mean (SSEM) for this statistic was calculated as $SSEM = \frac{{var\left( {F2} \right)}}{{n_{F2} }} + \frac{{\frac{{var\left( {BY} \right)}}{{n_{BY} }} + \frac{{var\left( {RM} \right)}}{{n_{RM} }}}}{4}$, where var(F2) is the variance of the F2 segregants’ metabolite levels, var(BY) is the variance of the BY replicates, var(RM) is the variance of the RM replicates, n_F2_ is the number of F2 segregants, and n_BY_ and n_RM_ are the number of replicates for the respective parents. Dividing the Δ- statistic by the square root of the SSEM yielded t-values for each of the metabolites. To assess significance, the parental and segregant levels were appended, the choice of “parent” measurements was randomized 1000 times, and the t-value was calculated. The Bonferroni-corrected threshold for significance was 0.05/8 due to the 8 metabolites tested, and so real t-values greater than the 99.375% of the absolute values of the permuted t-values were called significant.

## Supplementary Information

**Additional file 1: Fig. S1.** Technical Replicate Correlations for Remaining Metabolites, Scaled By All Segregants’ Mean Metabolite Levels. **A** Between technical replicate pearson correlation of squalene level for 73 F2 segregants with technical replicates. **B:** Between technical replicate pearson correlation of epoxysqualene level for 73 F2 segregants with technical replicates. **C:** Between technical replicate pearson correlation of lanosterol level for 73 F2 segregants with technical replicates. **D:** Between technical replicate pearson correlation of DCT level for 73 F2 segregants with technical replicates. **E:** Between technical replicate pearson correlation of DCD level for 73 F2 segregants with technical replicates. **F:** Between technical replicate pearson correlation of CDO level for 73 F2 segregants with technical replicates. **G:** Between technical replicate pearson correlation of Zymosterol level for 73 F2 segregants with technical replicates.

**Additional file 2: Fig. S2.** Metabolite Ratio Correlations. **A:** Correlations between the logarithm base two of metabolite ratios.

**Additional file 3: Fig. S3.** Overlap Between eQTLs and metabolite QTLs. **A:** Venn Diagram showing the overlap between eQTLs mapped for genes within the ergosterol bionsynthesis pathway and metabolite QTLs. **B:** Distribution of the number of permuted metabolite QTLs out of nineteen possible, overlapping ergosterol pathway eQTLs from 1000 permutations. Values greater than or equal to the true overlap from the data are in orange.

**Additional file 4: Fig. S4.** Permutation p-value distributions from the Kruskal–Wallis Test comparing Segregant and Parental Distributions of Metabolite Levels. **A:** Histogram of p-values from the Kruskal–Wallis test for permutations of Squalene levels. **B:** Histogram of p-values from the Kruskal–Wallis test for permutations of Epoxysqualene levels. **C:** Histogram of p-values from the Kruskal–Wallis test for permutations of Lanosterol levels. **D:** Histogram of p-values from the Kruskal–Wallis test for permutations of DCT levels. **E:** Histogram of p-values from the Kruskal–Wallis test for permutations of DCD levels. **F:** Histogram of p-values from the Kruskal–Wallis test for permutations of CDO levels. **G:** Histogram of p-values from the Kruskal–Wallis test for permutations of Zymosterol levels. **H:** Histogram of p-values from the Kruskal–Wallis test for permutations of Ergosterol levels.

**Additional file 5: Fig. S5.** Remaining F2 and Parental Distributions of Metabolite Levels. All Metabolite Levels Scaled by the Mean of the RM metabolite levels. **A:** Histogram of F2 segregants’ distribution of Squalene levels, with three biological replicate measurements of each of the parental strains (BY red, RM blue). **B:** Histogram of F2 segregants’ distribution of Lanosterol levels, with three biological replicate measurements of each of the parental strains (BY red, RM blue). **C:** Histogram of F2 segregants’ distribution of DCT levels, with three biological replicate measurements of each of the parental strains (BY red, RM blue). **D:** Histogram of F2 segregants’ distribution of CDO levels, with three biological replicate measurements of each of the parental strains (BY red, RM blue). **E:** Histogram of F2 segregants’ distribution of Ergosterol levels, with three biological replicate measurements of each of the parental strains (BY red, RM blue).

**Additional file 6:** Ergosterol Pathway Metabolite Names and Chemical Structures.

**Additional file 7:** Segregant Genotypes at Genomic Markers used in the study, from Albert et al (2018).

**Additional file 8:** Normalized Peak Metabolite Levels for F2 Segregants.

**Additional file 9:** All Metabolite QTL Mapped.

**Additional file 10:** Normalized Peak Metabolite Levels for Parental Strain Replicates.

**Additional file 11:** Strain Growth and Metabolite Extraction Information.

**Additional file 12:** Normalized and Unnormalized Peak Areas for Metabolite Levels.

## Data Availability

Additional file 6: Table S1 contains full and alternate names and molecular formulas for the metabolites discussed in this study. Additional file 7: Table S2 contains all genotypes at segregating markers for the F_2_ segregants used in this study from Albert et al. [33]. Additional file 8: Table S3 contains the metabolite measurements for all F_2_ segregants. Additional file 9: Table S4 contains all mapped QTL. Additional file 9: Table S4 contains the metabolite measurements for the parental strains. Additional file 10: Table S5 contains strain growth and handling information. Additional file 11: Table S6 contains the raw metabolite readings and normalized readings which are in Additional files 8, 9, 12: Tables S3, S5, S12.
