# Supplementary figures and images for: Divergent patterns of selection on metabolite levels and gene expression
Source: BMC Ecol Evol. 2021 Sep 29;21:185. doi: 10.1186/s12862-021-01915-5 (PMC8482673; doi:10.1186/s12862-021-01915-5)

Figure S1

A

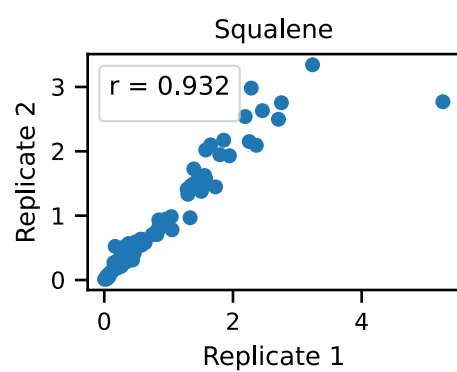

B

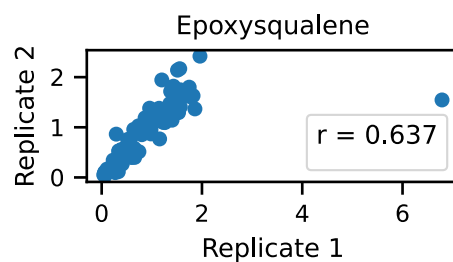

C

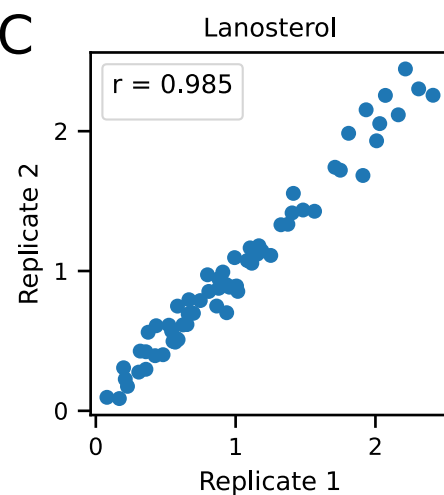

D

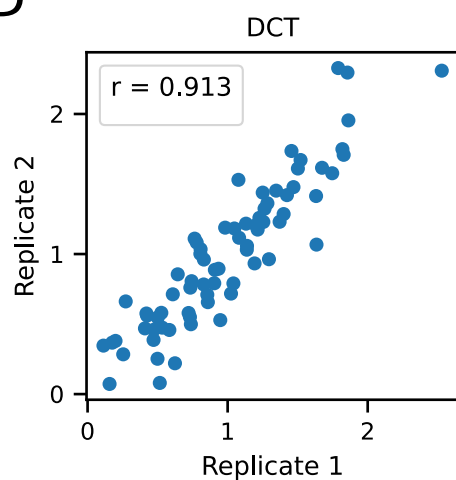

E

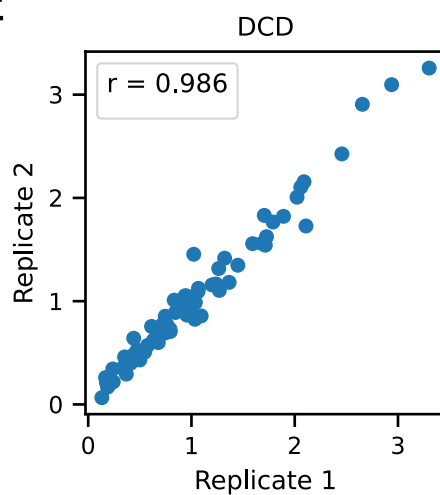

F

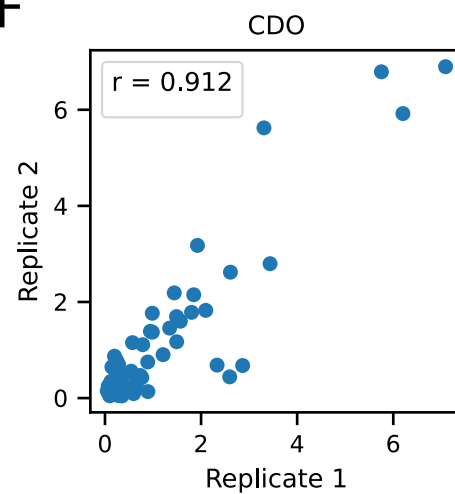

G

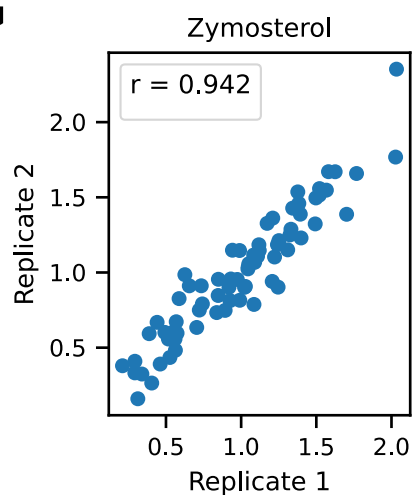

Supplement: Supplementary file 1 — Additional file 1: Fig. S1. Technical Replicate Correlations for Remaining Metabolites, Scaled By All Segregants’ Mean Metabolite Levels. A Between technical replicate pearson correlation of squalene level for 73 F2 segregants with technical replicates. B: Between technical replicate pearson correlation of epoxysqualene level for 73 F2 segregants with technical replicates. C: Between technical replicate pearson correlation of lanosterol level for 73 F2 segregants with technical replicates. D: Between technical replicate pearson correlation of DCT level for 73 F2 segregants with technical replicates. E: Between technical replicate pearson correlation of DCD level for 73 F2 segregants with technical replicates. F: Between technical replicate pearson correlation of CDO level for 73 F2 segregants with technical replicates. G: Between technical replicate pearson correlation of Zymosterol level for 73 F2 segregants with technical replicates. [file 12862_2021_1915_MOESM1_ESM.pdf]

Figure S2

A

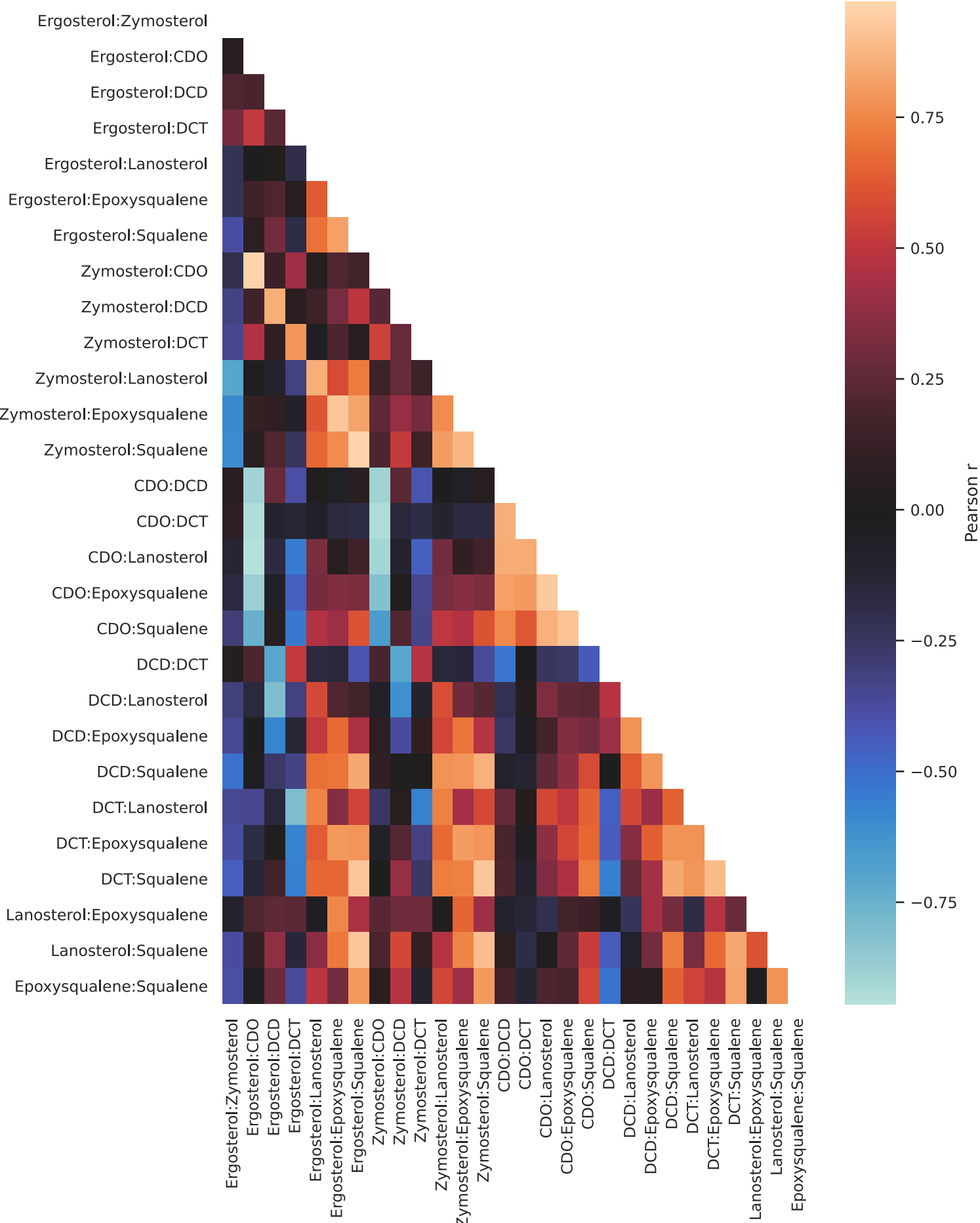

Supplement: Supplementary file 2 — Additional file 2: Fig. S2. Metabolite Ratio Correlations. A: Correlations between the logarithm base two of metabolite ratios. [file 12862_2021_1915_MOESM2_ESM.pdf]

Figure S3

A

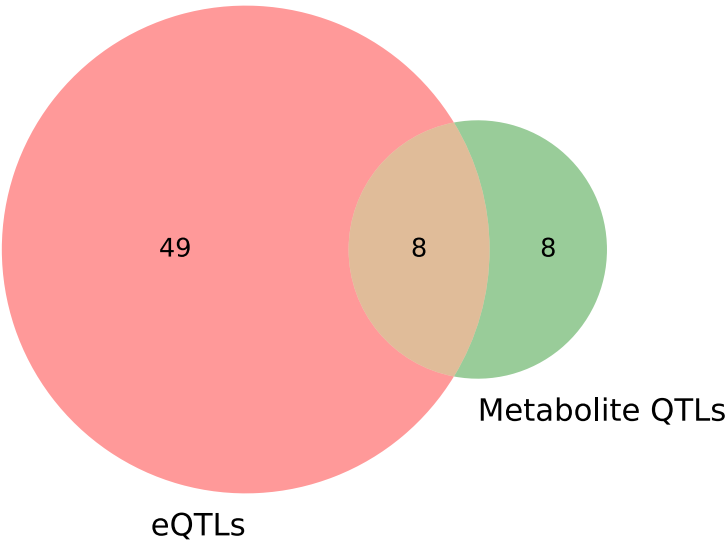

B

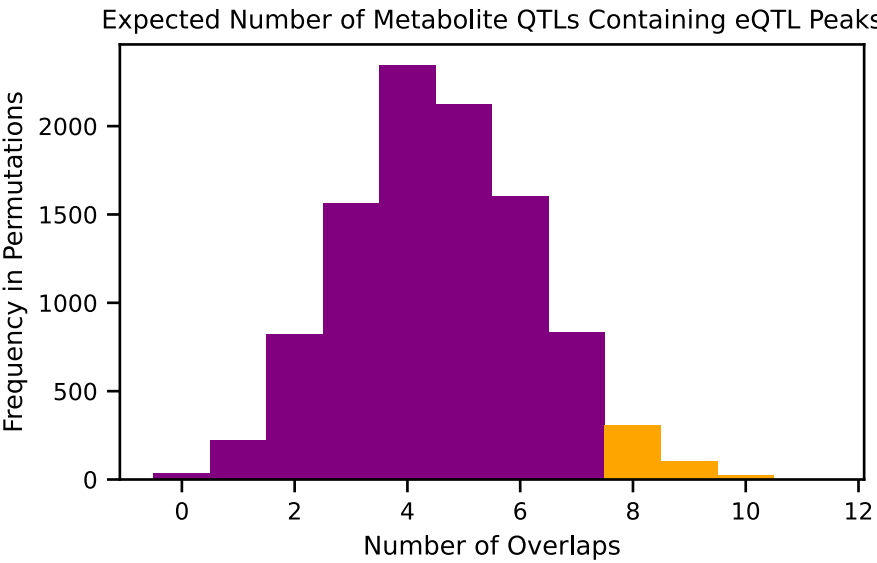

Supplement: Supplementary file 3 — Additional file 3: Fig. S3. Overlap Between eQTLs and metabolite QTLs. A: Venn Diagram showing the overlap between eQTLs mapped for genes within the ergosterol bionsynthesis pathway and metabolite QTLs. B: Distribution of the number of permuted metabolite QTLs out of nineteen possible, overlapping ergosterol pathway eQTLs from 1000 permutations. Values greater than or equal to the true overlap from the data are in orange. [file 12862_2021_1915_MOESM3_ESM.pdf]

Figure S4

A

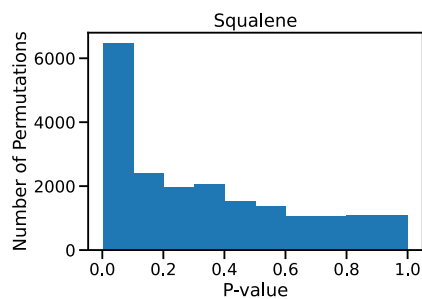

B

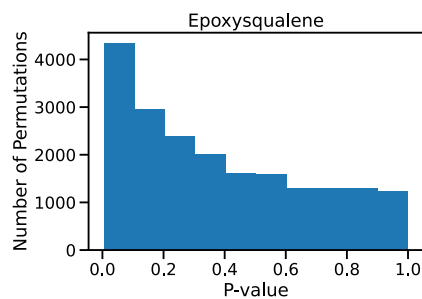

C

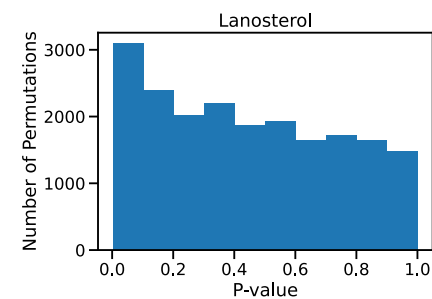

D

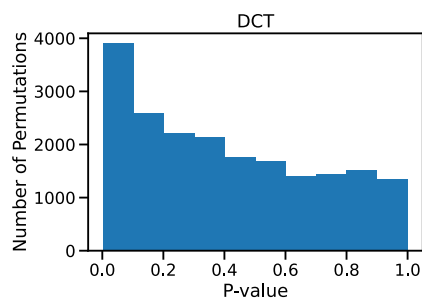

E

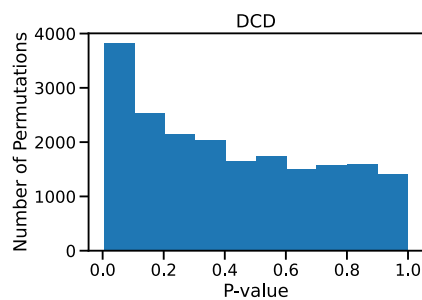

F

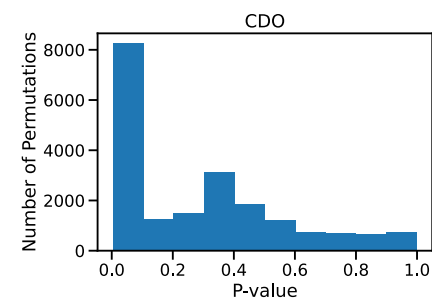

G

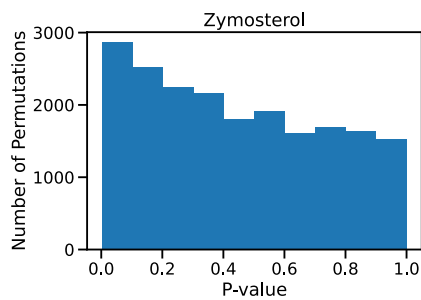

H

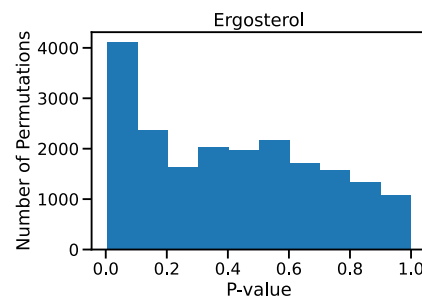

Supplement: Supplementary file 4 — Additional file 4: Fig. S4. Permutation p-value distributions from the Kruskal–Wallis Test comparing Segregant and Parental Distributions of Metabolite Levels. A: Histogram of p-values from the Kruskal–Wallis test for permutations of Squalene levels. B: Histogram of p-values from the Kruskal–Wallis test for permutations of Epoxysqualene levels. C: Histogram of p-values from the Kruskal–Wallis test for permutations of Lanosterol levels. D: Histogram of p-values from the Kruskal–Wallis test for permutations of DCT levels. E: Histogram of p-values from the Kruskal–Wallis test for permutations of DCD levels. F: Histogram of p-values from the Kruskal–Wallis test for permutations of CDO levels. G: Histogram of p-values from the Kruskal–Wallis test for permutations of Zymosterol levels. H: Histogram of p-values from the Kruskal–Wallis test for permutations of Ergosterol levels. [file 12862_2021_1915_MOESM4_ESM.pdf]

Figure S5

A

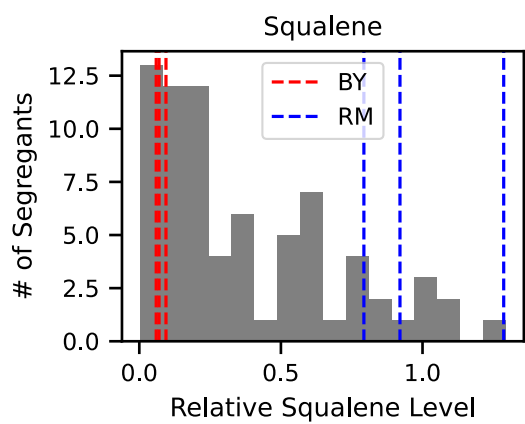

B

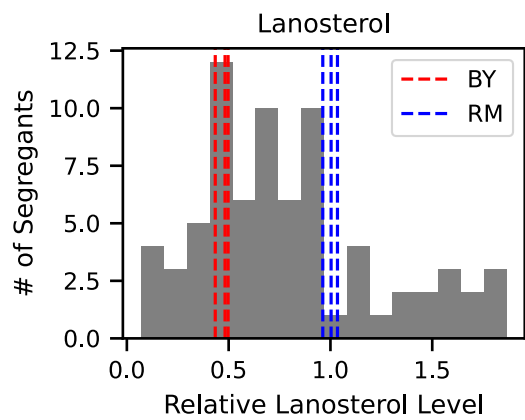

C

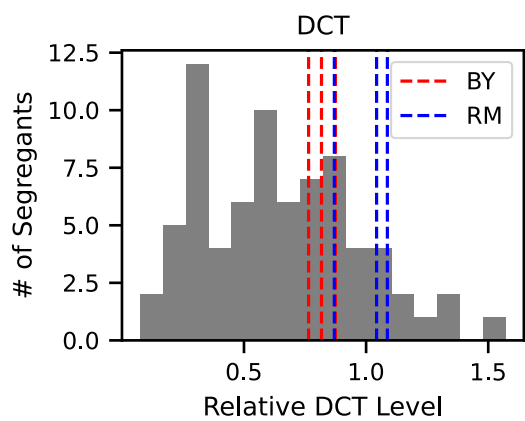

D

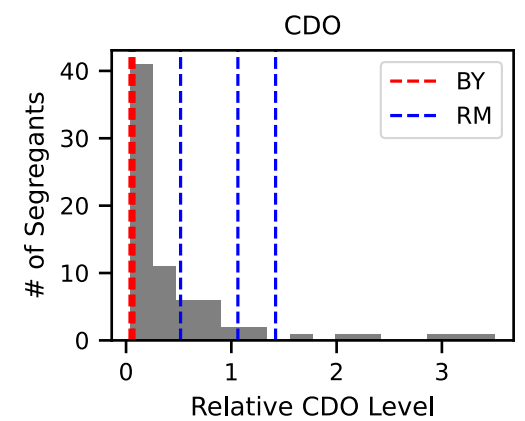

E

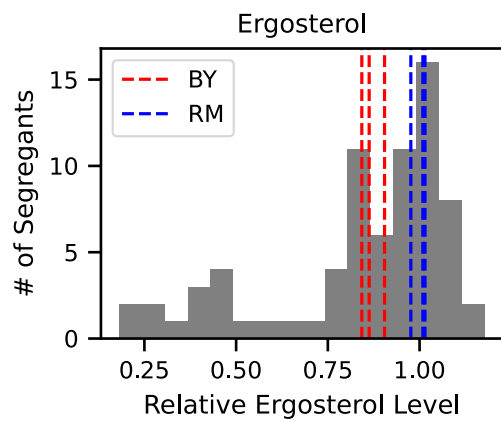

Supplement: Supplementary file 5 — Additional file 5: Fig. S5. Remaining F2 and Parental Distributions of Metabolite Levels. All Metabolite Levels Scaled by the Mean of the RM metabolite levels. A: Histogram of F2 segregants’ distribution of Squalene levels, with three biological replicate measurements of each of the parental strains (BY red, RM blue). B: Histogram of F2 segregants’ distribution of Lanosterol levels, with three biological replicate measurements of each of the parental strains (BY red, RM blue). C: Histogram of F2 segregants’ distribution of DCT levels, with three biological replicate measurements of each of the parental strains (BY red, RM blue). D: Histogram of F2 segregants’ distribution of CDO levels, with three biological replicate measurements of each of the parental strains (BY red, RM blue). E: Histogram of F2 segregants’ distribution of Ergosterol levels, with three biological replicate measurements of each of the parental strains (BY red, RM blue). [file 12862_2021_1915_MOESM5_ESM.pdf]
